# Removal of Ciprofloxacin and Norfloxacin from Aqueous Solution with Activated Carbon from Cupuaçu (*Theobroma grandiflorum*) Bark

**DOI:** 10.3390/molecules29245853

**Published:** 2024-12-11

**Authors:** Rafael Alves do Nascimento, Nilson dos Reis de Oliveira Novaes, Demetrius Pereira Morilla, Patricia Teresa Souza da Luz, Cristiane Maria Leal Costa, Lênio José Guerreiro de Faria

**Affiliations:** 1 Postgraduate Program in Amazonian Natural Resources Engineering, Federal University of Pará, Rua Augusto Correa, 01, Belém 66075-110, Brazil; lenio@ufpa.br; 2 Federal Institute of Education Science and Technology of Rondônia, Porto Velho Calama Campus, Av. Calama, 4985, Porto Velho 76820-441, Brazil; nilson.novaes@ifro.edu.br; 3 Federal Institute of Education Science and Technology of Alagoas, Maceió Campus, Rua Mizael Domingues, 530, Maceió 57020-600, Brazil; demetrius.morilla@ifal.edu.br; 4 Federal Institute of Education Science and Technology of Pará, Belém Campus, Av. Alm. Barroso, 1155, Belém 66093-020, Brazil; patricia.luz@ifpa.edu.br; 5 Postgraduate Program in Chemical Engineering, Federal University of Pará, Rua Augusto Correa, 01, Belém 66075-110, Brazil; cristianemlcosta@gmail.com; 6 Chemical Engineering School, Federal University of Pará, Rua Augusto Correa, 01, Belém 66075-110, Brazil

**Keywords:** fluoroquinolones adsorption, antibiotics pollution, cupuaçu bark, electrostatic interactions, regeneration of activated carbon

## Abstract

The widespread use of antibiotics such as fluoroquinolones (FQs) has raised environmental and health concerns. This study is innovative as we investigate the removal of ciprofloxacin (CIP) and norfloxacin (NOR) from water using activated carbon derived from cupuaçu bark (CAC). This previously discarded biomass is now a low-cost raw material for the production of activated carbon, boosting the local economy. CAC was physiochemically characterized, and adsorption experiments were designed using the Box–Behnken design to assess the effects of contact time, adsorbate concentration, and adsorbent dosage on the removal efficiency and adsorption capacity. The optimal conditions were determined using the desirability function, and kinetic, isothermal, and thermodynamic experiments were performed. CAC showed a 50.22% yield, low humidity (4.81%), and low ash content (4.27%), with acidic functional groups dominating. The surface area was 1335.66 m^2^/g, with an average pore volume of 0.753 cm^3^/g and a pore diameter of 2.206 nm. Adsorption was most effective at pH 5.0 due to electrostatic interactions between the basic adsorbent and cationic forms of CIP and NOR. Optimal conditions yielded adsorption capacities of 6.02 mg/g for CIP and 5.70 mg/g for NOR, with the Langmuir model suggesting monolayer adsorption. The regeneration with NaOH was effective, but the adsorption efficiency decreased below 50% after two cycles. These findings demonstrate that CAC is a sustainable, low-cost adsorbent for treating antibiotic-contaminated water.

## 1. Introduction

Aquatic contamination by pharmaceutical compounds is a significant environmental concern, with antibiotics being detected, over the last decades, in water matrices from hospital effluents and veterinary activities, as well as treatment plant sludge and wastewater samples [1]. Antibiotics are capable of inhibiting or destroying beneficial bacteria even in small concentrations, as well as bioaccumulating and biomagnifying in food chains, posing risks to higher trophic levels, including humans [2]. It is estimated that antibiotic-resistant infections cause approximately 700,000 deaths a year globally, indicating a growing and alarming problem [3].

Among the antibiotics identified in the aquatic environment, fluoroquinolones (FQs), such as ciprofloxacin (CIP) and norfloxacin (NOR), are frequently detected. These antibiotics can control broad-spectrum bacteria (Gram-positive and negative bacteria), which are the cause of serious and invasive infections, with CIP and NOR being widely used in human and veterinary medicine [4]. 

Several technologies have been developed as alternatives for treating wastewater containing these contaminants, such as membrane separation, electrochemical oxidation, and ozonation. However, such technologies are expensive and difficult to operate on an industrial scale [5]. In this context, adsorption stands out as an effective technology for removing these pollutants, offering low operating costs, high efficiency, and easy operation procedures. However, the selection of the adsorbent material is a crucial factor that interferes with the removal efficiency of the contaminant and the total cost [6].

Activated carbons (ACs) are widely used as adsorbents owing to their high adsorption capacity, increased porosity, and large surface area. The production of ACs requires the precursor to undergo a carbonization and activation process, which can be physical, using heat, or chemical, using chemical agents. Each method of activating activated carbon has specific characteristics that influence the production process and the final properties of the material [7]. Physical activation, for example, generates a predominantly microporous activated carbon, without the need for chemical additives, and requires high temperatures. In contrast, chemical activation uses lower temperatures and shorter activation times, with the aid of chemical agents that facilitate the opening of and increase in pores in the precursor material, generating an activated carbon with a mixture of micropores and mesopores [8].

With the objective of reducing the cost of this process, obtaining AC from agro-industrial waste is advantageous, since this waste is lignocellulosic biomass, which, in addition to converting it into an adsorbent, adds commercial value to the product and reduces the cost involved in the final waste disposal [9,10].

Cupuaçu is one of the most prominent native fruits in the Amazon, largely due to the endocarp (edible part) of the fruit, which is used to make the pulp. Yellowish white in color, the pulp of cupuaçu is preferred by consumers owing to its characteristic taste and pleasant smell, and among other food applications, it is used for making juices, sweets, jellies, and ice cream. Furthermore, the by-products resulting from the industrialization of cupuaçu pulp, such as bagasse and seeds, are used for developing other food products [11]. However, the peels (epicarp) have no industrial application and are usually used as a fertilizer, burning material for boilers and furnaces, or even discarded as organic waste [12]. 

Recent studies aim at using this residue on an industrial scale and adding value to this biomass through the development of chemical products and processes [13,14]. Being a rich source of lignocellulose, the cupuaçu peel has already been used as a precursor material for ACs for CO_2_ removal from the environment [15] and dye adsorption from water [16]. However, studies on the use of AC from cupuaçu bark to remove antibiotics are scarce, highlighting the pioneering nature of this study. The aim of this study was to investigate the removal of CIP and NOR from an aqueous solutions using AC produced from cupuaçu peel, an unconventional and low-cost adsorbent. A Box–Behnken design (BBD), combined with a simultaneous optimization technique, was conducted in order to evaluate the process variables and find the optimum adsorption conditions. Furthermore, the adsorption mechanism for CIP and NOR was investigated via adsorbent characterization analyses, kinetic and equilibrium experiments, and thermodynamic data, and the chemical regeneration of the adsorbent was conducted to verify its viability in the remediation of effluents.

## 2. Results and Discussion

### 2.1. Characterization of AC

#### 2.1.1. Yield, Moisture, and Ash

Cupuaçu bark (CAC) exhibited yield, moisture, and ash values of 50.22 ± 2.24%, 4.81 ± 0.15 g/100 g, and 4.27 ± 0.11%, respectively, with the yield being within the range reported in the literature for AC obtained via activation with H_3_PO_4_ (42.15 at 56.25%) at temperatures near 600 °C [17]. Compared to other residual Amazonian biomass such as murumuru endocarp, Pará nut hedgehog, and açaí kernels, with reported yields of 40.81% [18], 34.7% [19], and 55.0% [20], respectively. Our results are similar, indicating that the cupuaçu peels used in this study as a CAC precursor are an excellent carbonaceous biomass.

The low moisture content is a good indication that the carbonization process was efficient. Excess moisture in AC can influence the adsorption process, with water molecules occupying the AC pores instead of the adsorbate molecules, thus hindering pore–adsorbate interactions [21]. Moreover, the moisture content in CAC was close to that described in ASTM D2867-23 [22] for granular AC with these characteristics (4.9%).

CAC exhibited a low ash content value, which is entirely desirable. A high ash content (>12%) implies a decreased mechanical strength and adsorption capacity of the carbonaceous solid [21,23]. Furthermore, depending on the solution in which AC is immersed, the solubilization of ash can modify the pH of the medium and promote undesirable reactions [24,25]. 

#### 2.1.2. Surface Functional Groups, pH at the Zero-Load Point (pH_PZC_), and Surface pH 

Due to the activation process with H_3_PO_4_, a higher content of acidic groups was identified (3.982 ± 0.218 mg Eq/g) compared to the basic ones (0.092 ± 0.002 mg Eq/g). The contents of phenolic (-OH), carboxylic (-COOH), and lactone (-COOR) groups were 1.953 ± 0.095, 1.318 ± 0.083, and 0.711 ± 0.055 mEq/g, respectively, attributing a predominantly acidic characteristic to CAC.

Phenolic groups and lactones are considered relatively weak, dissociating at higher pH values than carboxylic acid groups [26]. A similar behavior was reported by Neme et al. [27], indicating that in the process of activation with H_3_PO_4_, the phosphate groups are introduced into the molecular chain of the biomass, promoting molecular interactions, resulting in the acidic character of the obtained AC.

**Figure 1:**
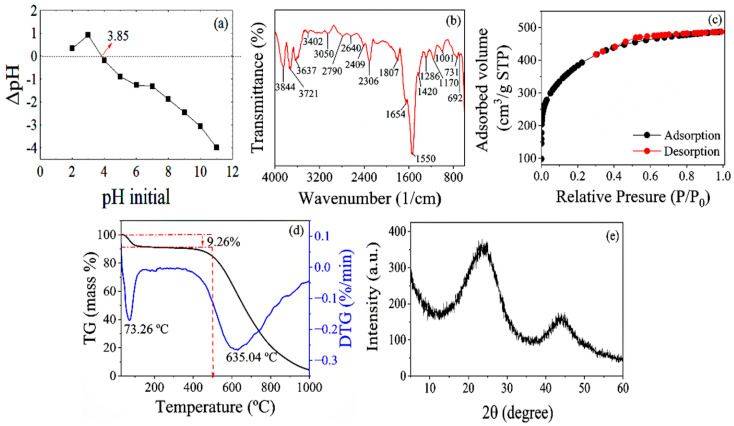
Zero charge point (pH_pcz_) (**a**), FTIR spectrum (**b**), N_2_ isotherms (**c**), TG and DTG (**d**) and XRD (**e**) of CAC.

CAC exhibited a pH_PZC_ (Figure 1a) value similar to that of other H_3_PO_4_-activated ACs, whose precursors were also biomass from the Amazon region, such as AC obtained from Pará nutshells and açaí seeds, with pH_PZC_ values of 3.9 and 3.6, respectively [28]. The pH_PZC_ value indicates the pH value at which CAC has zero electrical charge on its surface, i.e., the number of positive charges is equal to the number of negative charges [29]. Knowing the pH_PZC_ value of an adsorbent allows us to predict its surface charge according to the pH of the solution. For solution pH values above pH_PZC_, the surface of the adsorbent is predominantly negative (basic); therefore, the adsorbent will have an affinity for interacting with cationic species. Conversely, for solution pH values below pH_PZC_, the adsorbent will have an affinity for interacting with anionic species, as its surface is predominantly positively charged [30].

This parameter is extremely important for the study of adsorption, as it directly influences the interaction of the adsorbate ions with the charges of the adsorbents, making it possible to predict the electrostatic interactions (repulsion or attraction) and reveal whether the pH value of the solution may or may not favor the adsorption process.

#### 2.1.3. Fourier Transform Infrared Spectroscopy (FTIR) Analysis

The FTIR spectrum of CAC with absorption bands in the 400–4000 cm^−1^ range is presented in Figure 1b, revealing the functional groups present on its surface. The spectrum exhibits bands at 3844 and 3721 cm^−1^, attributed to fissile water molecules according to Zubir and Zaini [31]. Furthermore, the absorption bands at 3637, 3402, 3050, and 2790 cm^−1^ correspond to OH groups, especially free OH (3637 cm^−1^) and associated OH in phenols, which participate in intramolecular bonds (3402 cm^−1^), and OH that participate in intramolecular hydrogen bonding with C=O (3050 and 2790 cm^−1^), whose interactions are typical in carboxylic acids [32]. 

According to the literature, the bands between 2306 and 2640 cm^−1^ are attributed to OH groups present in carboxylic acids [31], while the 1807 cm^−1^ band is associated to the carbonyl group [33], suggesting the occurrence of carboxylic acids and lactones, confirming the analysis of surface functional groups. The most intense absorption band at 1550 cm^−1^ and the band at 654 cm^−1^ are related to C=C groups from aromatic rings, indicating the presence of phenolic compounds in addition to the CAC graphitic structure [27,28]. 

Furthermore, the bands at 1420 and 1286 cm^−1^ are related to the C-O group of carboxylic acids due to the coupling of the angular deformation in the plane of the O-H bond and the axial deformation of C-O [34,35]. The band at 1170 cm^−1^ is attributed to hydrogen-bonded P=O stretching and O-C stretching vibrations in aromatic P-O-C. The band at 1001 cm^−1^ is ascribed to the stretching of the P-O-C group (aliphatic), the asymmetric stretching of P-O-C (aromatic), the P-OH bending, the asymmetric stretching of P-O-P in polyphosphates, and the symmetric stretching of PO_2_ and PO_3_ in phosphate–carbon complexes. These functional groups are presumably derived from the carbon activation process with H_3_PO_4_ [36,37].

The bands at 731 and 692 cm^−1^, are indicative of the out-of-plane deformation of the C-H group in substituted benzene rings, suggesting the presence of phenolic groups [38]. Therefore, the FTIR results indicate the greater presence of acidic groups in CAC, confirming the analysis of functional groups.

#### 2.1.4. Specific Surface Area 

The determination of the specific surface area, total volume, and pore diameter of CAC was conducted via N_2_ adsorption/desorption tests using Brunauer–Emmett–Teller (BET) isotherms. The obtained specific surface area (S_BET_), V_pores_, and Dp values for CAC were 1335.66 m^2^/g, 0.753 cm^3^/g, and 2.206 nm, respectively, being in agreement with the surface areas (589–1651.31 m^2^/g) and total pore volumes (0.280–1.220 cm^3^/g) of other biomass-derived ACs, also chemically activated with H_3_PO_4_ (Table S1). 

The N_2_ adsorption–desorption isotherm of CAC is presented in Figure 1c. According to the IUPAC classification (1985), updated by Thommes et al. [39], the isotherm can be classified as Type I (b). This type of isotherm exhibits a concavity in relation to the relative pressure axis (P/P_0_) and the adsorbed amount tends to stabilize at a limit value. Generally, this indicates the formation of a single monolayer of adsorbate on the surface of the adsorbent. It can also be seen in Figure 1c that for low values of relative pressure (P/P_0_ ≈ 0.05), the volume of adsorbed gas does not start at zero. This “jump” in the volume of adsorbed gas at relatively small pressures suggests the presence of micropores [28]. 

CAC exhibited a Dp value of ~2.2 nm, confirming the isotherm classification as Type I (b), according to Thommes et al. [39]. These isotherms are common in materials with pore size distributions over a wider range, including wider micropores and possibly narrow mesopores (<~2.5 nm). Figure 1c shows that after filling the monolayer, the desorption curves exhibit a subtle increase in the mesoporous region, indicating a small hysteresis loop of type H4, which is characteristic of slit-shaped pores [40]. 

The high specific surface area and the significant pore volume of CAC (both obtained by the N_2_ adsorption/desorption method using the BET isotherm), together with the possible presence of a high volume of micropores and narrow mesopores (observed by the characteristic of the isotherm) may be related to the use of H_3_PO_4_ as an activating agent. According to Sych et al. [41] and Tareq et al. [42], H_3_PO_4_ acts as an acid catalyst, promoting the rupturing of bonds in the precursor, the formation of cross-links via cyclization, and condensation reactions. Furthermore, H_3_PO_4_ can also merge with organic species to form phosphate and polyphosphate bridges. The presence of these phosphate groups causes structural expansion, leaving the matrix in an enlarged state with a structure of accessible pores, justifying the large volume of pores and high specific surface area of CAC compared to other chemical activating agents, as observed in Table S1.

#### 2.1.5. Thermal Analyses

Figure 1d presents the thermogravimetric analysis (TG) curve and the derivative of the thermogravimetric analysis (DTG) of CAC. The first mass loss event (~9.26%) was observed between 50 and 100 °C and may be related to the loss of water molecules physiosorbed on the CAC surface, as reported by Yadav et al. [43]. Between 100 and 500 °C, minor variations in mass were observed, indicating the absence of the main structural components of the cupuaçú peel (hemicellulose, cellulose, and lignin) due to the carbonization process. Hemicellulose and cellulose exhibit degradation temperatures in the range of ~200–400 °C, while the relevant lignin range is between ~250–500 °C [44]. In this context, it can be stated that CAC is thermally stable up to ~500 °C because that is the carbonization temperature used in preparation.

The second mass loss event begins at 500 °C and corresponds to the degradation and volatilization of components of carbonaceous materials, such as tar and oxygenated surface groups, until complete carbonization [45]. The temperature corresponding to the maximum oxidation rate of CAC was identified at ~635 °C and is close to that of other lignocellulosic ACs activated with H_3_PO_4_ under similar conditions, such as 613 °C for CA obtained from elderberry inflorescence [46], ~675 °C for CA obtained from sugar cane bagasse [47], and 617 °C for CA obtained from coconut shells [48].

#### 2.1.6. X-Ray Diffraction (XRD) and Morphology

The XRD spectrum of CAC is shown in Figure 1e, containing two broad and diffuse reflections of different intensities at approximately 2θ = 24° and 44°, which are attributed to the amorphous character of CAC. In fact, ACs comprise graphitic materials, exhibiting broad reflections due to the formation of disordered carbon structures [49]. Typically, such broad regions are identified in the same angular range, regardless of the precursor and activating agents used in the production process [18,28].

**Figure 2:**
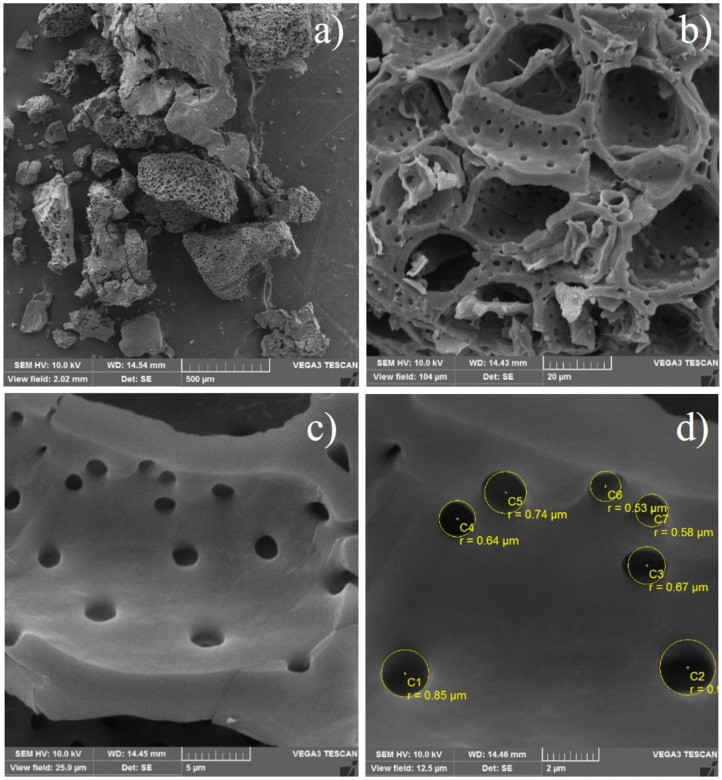
SEM images of CAC at different magnifications: (**a**) 0.1 k, (**b**) 2.0 k, (**c**) 4.0 k, and (**d**) 16.6 k.

The scanning electron microscopy (SEM) images of CAC are shown in Figure 2, clearly exhibiting a porous structure with a dense and irregular texture (Figure 2a). Furthermore, a tubular structure was observed in some parts (Figure 2b), indicating a possible preservation of the anatomy of the cellular tissue of the precursor after activation and carbonization, as proposed by Hu et al. [50]. Pores with spherical characteristics were identified (Figure 2c) with diameters ranging from 0.53 to 0.85 μm (Figure 2d). These characteristics of CAC can facilitate mass transfer from the adsorbate to the adsorbent, since the adsorbate molecules can easily reach the interior of the structure, facilitating its adsorption into the internal structures [51].

### 2.2. Preliminary Tests 

**Figure 3:**
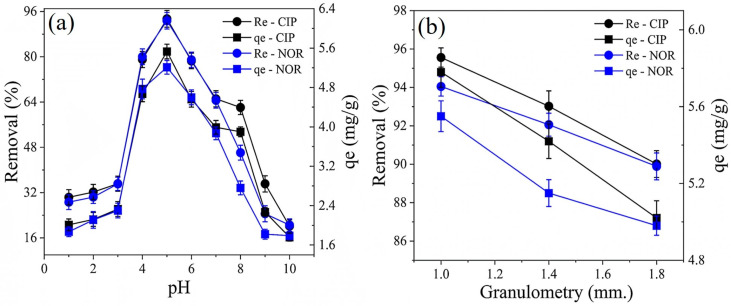
Effect of varying the pH of the solution (**a**) and particle size (**b**) on the removal and adsorption capacity of CIP and NOR by CAC.

The pH of the solution is an essential parameter influencing the adsorptive process. An unsuitable pH value can modify the surface charges on the CA surface and promote the dissociation of the adsorbate into anionic and/or cationic species, as well as harm any electrostatic interactions between the two. Figure 3a shows the removal and adsorption capacity of CIP and NOR by CAC as a function of pH alterations.

CAC exhibited similar adsorption properties (*qe* (mg/g) and *Re*(%)) for both antibiotics tested in this study. For pH values ranging from 4.0 to 6.0, higher *Re* and *qe* values were observed (Figure 3a), with the removal of CIP and NOR varying from ~80% (pH = 4.0), reaching its maximum at ~93% (pH = 5.0), and returning to 80% at pH = 6.0. The adsorption capacity also exhibited the same behavior with variations of ~4.7, 5.4, and 4.6 mg/g at pH values of 4.0, 5.0, and 6.0, respectively. 

**Figure 4:**
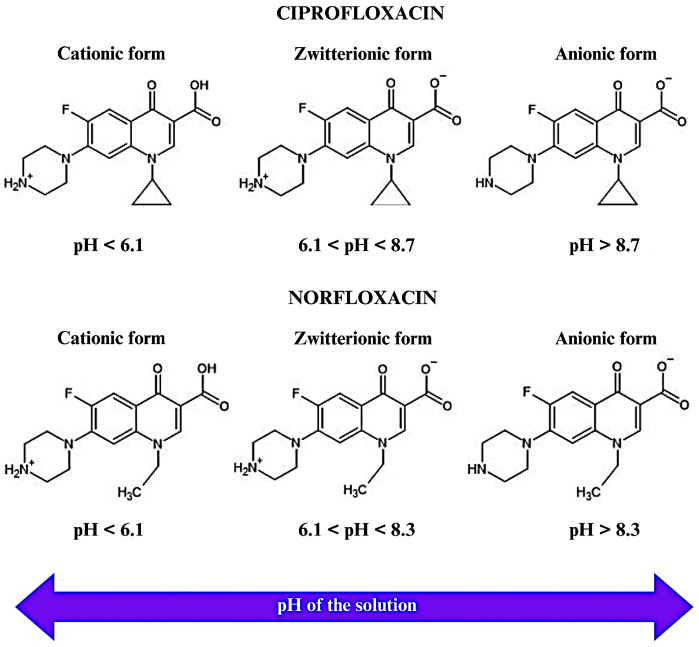
Chemical forms of CIP and NOR as a function of pH.

CIP and NOR are zwitterionic compounds as shown in Figure 4 (CIP: pka_1_ = 6.1 and pka_2_ = 8.7; NOR: pka_1_ = 6.1 and pka_2_ = 8.3). Depending on the pH of the solution, they dissociate and are present in their cationic or anionic forms [2]. At pH < 6.1, CIP dissociates in its cationic form with a protonated amine group, whereas at pH > 8.7 it dissociates in its anionic form with the deprotonation of its carboxylic acid. In turn, NOR is predominantly cationic at pH < 6.1 and anionic at pH > 8.3. 

CAC mainly contains acidic groups on its surface, thus modifying the pH of the solution and providing chemical modifications to the functional groups of the adsorbates, altering their charges and facilitating the adsorptive phenomena. Therefore, CIP and NOR in their anionic or cationic forms, or even antibiotics in their electrically neutral forms, can interact electrostatically with the functional groups of CAC.

In solutions with a pH below the pH_PZC_ of CAC (3.85), positive charges appear on the CAC surface due to the protonation of the basic groups and protonation of the carbonyls present in the carboxylic acids. Thus, for pH values < 3.85, CAC will exhibit an acidic character with an affinity for anionic species. According to Guilarduci et al. [52], phenolics and lactones hydrolyze at pH values close to 10 and 7, respectively. Therefore, the carboxylic acids present in CAC are the functional groups that probably undergo chemical modifications and consequently influence the surface charges of CAC. For pH values >3.85, negative charges form on the CAC surface due to the deprotonation of the carboxylic acids, which according to Martínez et al. [53], occurs at pH values close to 4, followed by the deprotonation of the lactone and phenolic groups. Thus, the CAC surface will have a basic character and therefore, affinity for cationic species. In this context, the pH region between 4.0 and 6.0 is extremely favorable for electrostatic interactions between the CIP and NOR ions and the surface charges of CAC, as the CIP and NOR molecules are predominantly in their cationic forms with their amine group protonated, and the carboxyl groups present in CAC are deprotonated. This condition promotes electrostatic interactions between the functional groups, corroborating the results shown in Figure 3a, where the highest values of *Re*(%) and *qe* (mg/g) are observed in the pH range between 4.0 and 6.0, with the maximum value being observed at pH 5.0.

At a solution pH < 3.85 (pH_PZC_ of CAC) the CAC surface is positively charged and CIP and NOR are predominantly in their cationic form. Therefore, electrostatic repulsions occur between the protonated functional groups of CAC and the protonated amine groups of the two antibiotics. This behavior confirms the analysis in Figure 3a for pH values from 1.0 to 3.0, with CIP and NOR exhibiting *Re*(%) and *qe* (mg/g) values for CAC lower than 35% and 2.8 mg/g, respectively. In the pH range of 6.1 to 8.3, both antibiotics are in their zwitterionic form, that is, electrically neutral, and therefore, the electrostatic interaction process is not at its maximum capacity. Under this condition, the removal efficiency varied from ~20 to 80%. 

In the pH region from 8.3 to 8.7, CIP is in its zwitterionic form, NOR in its anionic form, and the CAC surface is negatively charged. In this situation, electrostatic repulsions occur between the anionic form of NOR and CAC, while CAC exhibits partial affinity for CIP. This behavior was also observed in Figure 3a, where CIP exhibited better *Re*(%) and *qe* values than NOR in this pH range. For pH values > 8.7, CIP and NOR are in their anionic forms and the surface charge of CAC is negative; therefore, no electrostatic interactions occur and the values of *Re*(%) and *qe* are low.

Figure 3b presents the adsorption results as a function of CAC particle size, revealing that the best values for *Re*(%) and *qe* (mg/g) were obtained when CAC had a smaller grain size of 1.0 mm. According to Belhacemi [54], ACs with smaller particle sizes have a higher number of active sites and therefore, favor the phenomenon of adsorption. Such a finding has been also reported by Othmani et al. [55] and Pathak et al. [56] in studies on the removal of pollutants in aqueous solutions, indicating that the decrease in AC particle size implies a greater contact surface between the adsorbent and the adsorbate, optimizing the sorption of the adsorbate molecules. De Oliveira Brito et al. [57] and Ferreira et al. [58] also confirmed this conclusion, indicating that the larger the size of the adsorbent particles, the greater the diffusive resistance this material suffers in the environment, thus interfering with adsorption.

### 2.3. Experimental Design

The adsorption tests were conducted in batches and the results of the experimental tests are presented in the BBD experiment matrix (Table S2). The modifications proposed by the planning provided variations in *Re*(%) and *qe* (mg/g) for both adsorption systems (CIP-CAC and NOR-CAC).

The results were statistically analyzed using analysis of variance, ANOVA (Table 1), and the significance of each main effect on the response variables was evaluated and calculated. Furthermore, the regression coefficients necessary for the construction of the second-order polynomial models were obtained, which can be viewed in Equations (1)–(4).

(1) $$qe\left(mg/g\right)=5.813+{0.356X}_{1}+{0.325X}_{2}-{0.284X}_{3}-0.269{X_{1}}^{2}-0.217{X_{2}}^{2}-\;0.194{X_{3}}^{2}+0.167X_{1}X_{2}-0.160X_{1}X_{3}-0.042X_{2}X_{3}$$

(2) $$Re\left(\%\right)=95.287+6.067X_{1}-9.655X_{2}+5.230X_{3}-11.466{X_{1}}^{2}-6.541{X_{2}}^{2}-\;11.266{X_{3}}^{2}-6.165X_{1}X_{2}+2.385X_{1}X_{3}-2.250X_{2}X_{3}$$

(3) $$qe\left(mg/g\right)=5.543+0.385X_{1}+0.291X_{2}-0.309X_{3}-0.210{X_{1}}^{2}-0.233{X_{2}}^{2}-\;0.203{X_{3}}^{2}+0.160X_{1}X_{2}-0.190X_{1}X_{3}-0.102X_{2}X_{3}$$

(4) $$Re\left(\%\right)=95.993+6.541X_{1}-9.689X_{2}+5.155X_{3}-11.468{X_{1}}^{2}-6.378{X_{2}}^{2}-\;11.750{X_{3}}^{2}-5.582X_{1}X_{2}+2.080X_{1}X_{3}-1.790X_{2}X_{3}$$

(5) $$X_{1}=\frac{T-240}{60}$$

(6) $$X_{2}=\frac{C_{ant}-180}{150}$$

(7) $$X_{3}=\frac{C_{CAC}-0.6}{0.2}$$

The polynomial models reproduced more than 98% of the experimental data within the studied operational conditions since the determination coefficients were ≥0.9832. Residual analysis also confirmed the goodness of fit. The residuals were independent, randomly distributed, and strictly followed the statistical requirements of normality and homoscedasticity.

#### 2.3.1. Influence of Operating Conditions on CIP and NOR Removal

**Figure 5:**
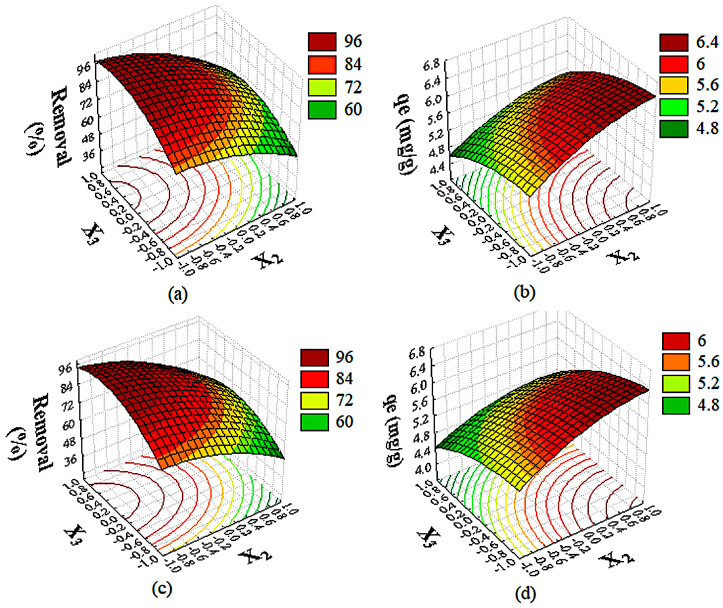
Response surface for removal and adsorption capacity at *X*_1_ = +1. CIP-CAC (**a**,**b**) and NOR-CAC (**c**,**d**).

The individual variables *X*_1_, *X*_2_, and *X*_3_ presented in Table 1 exhibited significant effects on the *Re*(%) of CIP and NOR, with *p* values ≤ 0.05. The response surface graphs (Figure 5a,c) show the behavior of *Re*(%) of CIP and NOR, respectively, as a function of changes in contact time (*X*_1_), CAC dosage (*X*_3_), and initial concentration of antibiotics (*X*_2_).

The analysis of the response surfaces revealed a similar removal percentage for both drugs using CAC as an adsorbent. A decrease in the initial concentration of CIP and NOR (variable *X*_2_) and an increase in the adsorbent dosage (variable *X*_3_) afforded a higher percentage of removal of CIP and NOR. Thus, the decrease in antibiotic concentration from 330 g/L (Level +1) to 30 g/L (Level −1), combined with the increase in CAC dosage from 0.4 g/L (−1) to 0.8 g/L (+1) resulted in removal percentages greater than 90%, as seen in Figure 5a,c.

The initial concentration of the adsorbate is one of the main variables affecting the removal of antibiotics by AC and its negative effect on *Re*(%) has been already reported in several previous studies [59,60]. This behavior is associated with the saturation of the active sites of the adsorbent with adsorbate ions and their interaction process, either physical or chemical [61].

At lower initial concentrations of antibiotics, the ions occupy/interact with the active sites of CAC in an almost ideal proportion, affording a lower residual concentration of antibiotics in the solution after adsorption. However, at higher initial concentrations, physical and/or chemical interactions also occur, with the number of antibiotic ions to be adsorbed being much higher than the available sites, resulting in lower removal percentages [62].

The Brazilian legislation does not include any limits regarding the release of industrial or domestic effluents to ensure that surface or underground water bodies are not contaminated by antibiotics. However, fluoroquinolones have already been detected in concentrations ranging from 10 ng/L to 100 μg/L and 100 mg/L to 500 mg/L in wastewater and hospital effluents, respectively [2,63]. Therefore, the high CIP and NOR removals (greater than 90%) in concentrations above 30 g/L achieved in this study highlight the reliability and excellent efficiency of CAC, being highly promising for removing such compounds.

The positive influence of the adsorbent dosage (*X*_3_) on the percentage of drug removal can be also observed in Figure 5a,c. An increase in the CAC dosage from 0.4 to 0.8 g/L afforded a higher percentage of CIP and NOR removal (>90%). According to Cheikh et al. [64], the adsorbent dosage is another key factor in adsorption studies, and its influence on contaminant removal is associated with the greater availability of active sites capable of interacting with the adsorbate ions. At a higher concentration of CAC, there are more active sites capable of interacting with the CIP and NOR ions, rendering adsorption more efficient. Nguyen et al. [45] and Yang et al. [65] reported similar results, confirming the positive influence of the adsorbent dosage on the removal of CIP and NOR.

#### 2.3.2. Influence of Operating Conditions on CIP and NOR Adsorption Capacity

Based on ANOVA, the individual variables *X*_1_, *X*_2_, and *X*_3_ had an influence on the response variable (*p* ≤ 0.05). A similar behavior was observed for the CAC adsorption capacity considering both drugs as adsorbates. The increase in the initial concentration of CIP and NOR (variable *X*_2_) and the decrease in the adsorbent dosage (variable *X*_3_) afforded greater adsorption capacities. Thus, the increase in the initial concentration of antibiotics from 30 g/L (−1) to 330 g/L (+1), combined with the decrease in the CAC dosage from 0.8 g/L (+1) to 0.4 g/L (−1), resulted in adsorption capacities of approximately 6.0 mg/g, as seen in Figure 5b,d.

The effect of an increased initial concentration of antibiotics on enhancing the adsorption capacity can be attributed to the greater availability of the adsorbate ions for interactions with the active sites of AC. According to Avci et al. [66], at higher adsorbate concentrations, the active sites of the adsorbent have a greater capacity and ease of interaction, leading to an increase in the intensity of the driving force in mass transfer, considering the same number of active sites available in the process. Guo and Wang [59] and Cheikh et al. [64] reported similar results for the adsorption of CIP and NOR.

The negative influence of the adsorbent dosage on the adsorption capacity of CIP and NOR (Figure 5b,d) has been also reported in previous studies [45,67]. This behavior may be associated with the occupation/interaction of the active sites with the adsorbate ions. At a lower adsorbent dosage, the active sites are more easily filled due to the greater availability of the adsorbate ions for each adsorbent particle [63]. 

Furthermore, at higher adsorbent dosages, the active sites may overlap, and/or the carbon particles may aggregate, which would result in a decrease in the specific surface area and an increase in the length of the diffusional path [18]. Such an effect would impede interactions between the CIP and NOR ions with the CAC binding sites, impacting the non-filling or semi-filling of active sites, thus resulting in a decrease in the adsorption capacity.

### 2.4. Simultaneous Optimization of Responses

The simultaneous optimization of the studied responses (*qe* and *Re*(%) for both antibiotics) was performed using the global desirability function [68]. Table S3 presents the parameters used in the numerical optimization to simultaneously obtain the highest percentages of antibiotic removal and greater adsorption capacity.

Table 2 presents the best conditions, the optimized response values (predicted by the desirability function), as well as the experimental values obtained. According to Lazić [69], the adjustment of the function was considerably acceptable and excellent, since the global desirability coefficient is in the range of 0.8–1.0. The proximity of experimental and predicted values confirms the validity and efficiency of optimizing CIP and NOR adsorption in CAC.

### 2.5. Kinetics and Mass Transfer

The pseudo-first-order (PPO), pseudo-second-order (PSO), and Elovich models were applied to elucidate the adsorption kinetics of CIP and NOR on CAC. The results of the adjustments are summarized in Table 3, together with the estimated parameters and statistics.

**Figure 6:**
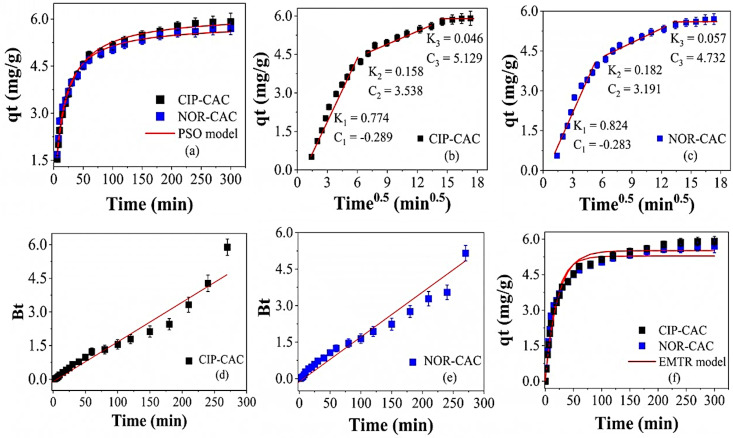
PSO models fit (**a**), intraparticle diffusion (**b**,**c**), Boyd (**d**,**e**), and EMTR (**f**) for the adsorption kinetics data.

The PSO model exhibited the best fit to the experimental kinetic data for the adsorption of CIP and NOR on CAC, affording the highest *R*^2^ and *R*^2^*_adjust_* values, ≥0.96 and ≥0.996, respectively, and the lowest Akaike information criterion (*AICc*) and root mean square error (*RMSE*) values, ≤−86.69 and ≤0.113, respectively. Furthermore, the PSO model also provided the best estimate of the equilibrium adsorption capacity (*qe*). The *qe* values afforded by this model are the closest to the experimental values, as presented in Figure 6a.

CIP adsorption on CAC ($Half-line(t_{1/2})$ = 18 min; Initial adsorption rate (h) = 0.3428 mg/g min) and NOR on CAC ($t_{1/2}$ = 14.21 min; h = 0.4123 mg/g min) exhibited similar speed characteristics, with relatively low $t_{1/2}$ values and high h values. Such results are in accordance with those reported by Rocha et al. [70] and Saucier et al. [71], who revealed that the investigated adsorbates in aqueous solutions present high initial adsorption rates and a relatively short half-life, probably due to the porosity and/or heterogenicity of the adsorbent surface. 

In the studies of CIP adsorption on AC with coconut biomass as a precursor [72] and desilicated rice husk residues [67], the PSO model was also the best fit for the experimental kinetic data. Similar behaviors were also observed in the adsorption of NOR on AC derived from rice husk and coffee [73] and lotus stems [74]. 

Kinetic models based on mass transfer by diffusion (intraparticle diffusion, Boyd diffusion, and EMTR) were also fitted to the experimental data to elucidate the mechanism of mass transfer between the adsorbates (CIP and NOR) and the adsorbent (CAC). Figure 6b,c show the fit of the intraparticle diffusion model to the experimental kinetic data for CIP and NOR, respectively, as well as the parameters obtained via the linear extrapolation of the fitted lines.

Multilinearity is observed, suggesting that the mechanism of adsorption of CIP and NOR on CAC has three stages. Section I corresponds to external surface adsorption or adsorption on the film. In this stage, the CIP and CAC ions transfer from the solution to the surface of the adsorbent and are rapidly adsorbed due to the high driving force. Section II corresponds to intraparticle diffusion. In this stage, the CIP and NOR ions that are adsorbed on the surface of CAC enter the pores and diffuse into the internal structure of the adsorbent. This is a slow process; therefore, the intraparticle diffusion constant *K_2_* is lower than *K*_1_. The value of C (intercept of the linear fit), which indicates the thickness of the boundary layer, does not intercept the origin, and therefore, intraparticle diffusion alone is not the controlling step in the adsorption rate [75]. Section III becomes evident when the number of available active sites becomes small and/or the CIP and NOR molecules exist in small quantities in the solution. Thus, the driving force is not enough to maintain the mass transfer and the adsorption reaches the equilibrium stage [76].

The plot of *Bt* versus *t* describing the Boyd diffusion model for the adsorption of CIP and NOR on CAC is shown in Figure 6d,e. None of the linear fits crosses the origin and the experimental points are scattered, indicating that the adsorption mechanism is governed by external diffusion or by external and intraparticle diffusion [77]. Figure 6f shows the fit of the EMTR model to the experimental kinetic data for CIP-CAC and NOR-CAC to verify whether diffusion in the outer film is the controlling step in the adsorption process. The external film diffusion model showed satisfactory fits with R^2^ values ≥ 0.986 and low RMSE values, indicating the model’s adequacy to explain ~98.8% of the experimental variability. 

The lowest K_TM_ value was observed for the CIP-CAC system, suggesting a greater resistance to mass transfer in the external film compared to the NOR-CAC system. This analysis is in agreement with the interpretation obtained from the intraparticle diffusion model, where the highest C value (a parameter that provides an understanding of the boundary layer thickness) was observed in the CIP-CAC system, indicating a thicker boundary layer and therefore, greater resistance to mass transfer. The EMTR model presented the best fit to the experimental data among the tested mass transfer models, indicating that for CIP-CAC and NOR-CAC, external mass diffusion was the controlling step in the adsorption process. Similar results were observed by Antonelli et al. [63] in the adsorption of CIP on modified clay and Liu et al. [26] in the adsorption of NOR on granular CA, in which external film diffusion was the dominant adsorption step.

### 2.6. Adsorption Equilibrium 

The fits of the Langmuir, Freundlich, and Sips isotherms to the experimental data of CIP-CAC and NOR-CAC at different working temperatures (28, 35, and 45 °C) indicated that the Sips and Langmuir models exhibited satisfactory fits, as detailed in Table 4. 

Both models exhibited the lowest *RMSE* and *AICc* values and consistently afforded the highest *R*^2^ and *R*^2^*_adjus_* values at all evaluated temperatures, with both being close to unity (≥0.96). However, the overestimated value of the maximum adsorption capacity of the monolayer (*q_m_*) fitted by the Sips model rendered it inadequate to represent the experimental data of the studied adsorption systems. Therefore, the Langmuir isotherm was adopted to represent the experimental adsorption equilibrium data for CIP-CAC and NOR-CAQ. 

The experimentally obtained adsorption isotherms of CIP and NOR on CAC (Figure S1a,b) can be classified as Type L2, according to the classification of Giles et al. [78], indicating the formation of a monolayer of adsorbate on the surface of the adsorbent. This result is consistent with the high affinity observed between the antibiotics and the CAC surface, particularly at low equilibrium concentrations (Figure S1a). At higher concentrations, the isotherms reached a maximum capacity, suggesting an adsorption saturation.

The Langmuir model was adequate to describe the experimental data, indicating the formation of an adsorbate monolayer and that all the active sites of CAC are energetically equal, as well as the absence of interactions between the adsorbate molecules [79]. Similar results have been reported from previous studies on the adsorption of CIP and NOR on AC made from bamboo biomass [59] and discarded coffee powder [45].

Table 4 shows that the experimentally obtained $q_{m}$ increased with increasing temperatures for both adsorption systems. This behavior was only predicted by the Langmuir model, further confirming the adequacy of this model to describe the CIP and NOR adsorption equilibrium data. Table S4 shows the values of the separation factor (*R*_*L*_) calculated at all initial concentrations and at all adsorption temperatures. The *R*_*L*_ values range from 0 to 1, indicating that the CIP and NOR adsorption isotherms are favorable, exhibiting a significant affinity of the adsorbate for the adsorbent, until equilibrium is reached [80].

The adsorption capacity values of CAC for CIP and NOR were compared to other adsorbent materials (Table S5), indicating that CAC is a promising adsorbent for removing fluoroquinolones, especially CIP and NOR. Compared to other adsorbents, CAC showed a similar or higher efficiency, possibly due to its large surface area, well-developed porous structure, and specific chemical affinity toward the CIP and NOR molecules.

### 2.7. Adsorption Thermodynamics

The thermodynamic parameters were obtained by calculating the equilibrium constant $K_{e}^{0}$ (dimensionless) using the Langmuir constant *K_L_* (L/mg) obtained from the adsorption equilibrium adjustments. The resulting $K_{e}^{0}$ values are presented in Table 5. Entropy (ΔS°) and enthalpy (ΔH°) variations, calculated using the Van’t Hoff equation (Equation (18)) in its linear form, along with the ΔG° values, are shown in Table 5.

The negative ∆G° values indicate that the adsorption process of CIP and NOR on CAC is favorable [79]. Similar results have been previously reported for the adsorption of CIP on AC from *Prosopis juliflora* [81] and the adsorption of NOR on magnetized bio coal [82]. An increase in temperature favored the adsorption process, since the negativity of the ∆G values increased with increasing temperature, as shown in Table 5. This result corroborates the analysis of the equilibrium study, where a greater adsorption capacity was achieved at a higher temperature.

The positive ΔS values (101.18 and 116.41 J/mol K) indicate an increase in randomness at the solid–solution interface in the CIP adsorption process. As adsorption and desorption occur naturally during the adsorption process, the increase in adsorbate molecule randomness is related to dissociative processes, in which the energy absorbed during bond and/or interaction breaking (desorption) is greater than the energy released during bond and/or interaction formation (adsorption) [79,83]. Given this, the positive ΔS values indicate that CIP and NOR have a high affinity for CAC, as the energy required to form bonds and/or interactions with CAC is lower than the energy required to break the same bonds and/or interactions. 

The positive enthalpy ΔH (9.78 and 7.37 kJ/mol) reveals an endothermic process, and the magnitude of ΔH indicates that the adsorption of CIP and NOR on CAC involves physisorption, since the ΔH values for CIP and NOR are <40 kJ/mol [83]. Previous studies reported a similar behavior, with the obtained thermodynamic parameters indicating that the adsorption of CIP and NOR was favorable, endothermic, and with a predominance of physisorption [81,82].

### 2.8. Adsorption Mechanisms of CIP and NOR on CAC

Understanding a potential adsorption mechanism requires the combination of various pieces of information obtained during the study. Assuming an adsorption mechanism based on adsorption kinetics or equilibrium analysis alone is incorrect [84]. A more effective analysis requires information on the surface chemistry of the adsorbent and its interactions with the adsorbate (pH_PZC_, Boehm titration, FTIR), an analysis of the textural and morphological properties of the adsorbent (S_BET_, XRD, SEM, and TGA), as well as information obtained from kinetic, equilibrium, and thermodynamic adsorption studies.

**Figure 7:**
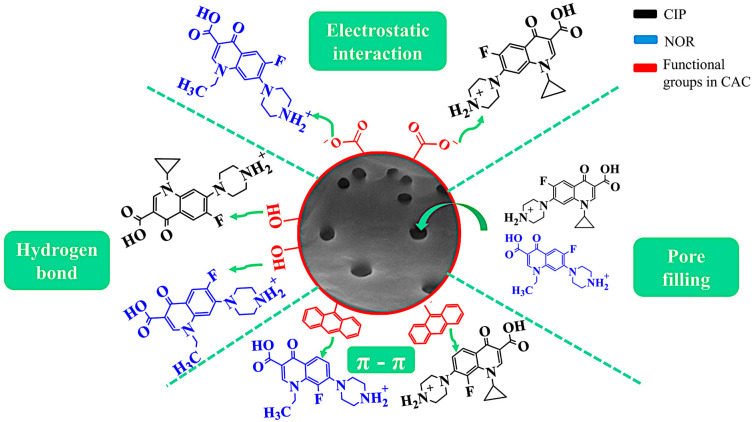
Scheme of the potential adsorption mechanism of CIP and NOR on CAC. The green arrows indicate the potential interactions between the functional groups (in red) of the CAC and the CIP (in black) and NOR (in blue) molecules.

Acid functional groups were the main groups identified on CAC via Boehm titration, with phenolic groups being predominant, followed by carboxylic groups and lactones. FTIR analysis corroborated these findings, revealing characteristic absorptions at 2640, 2306, 1807, 1654, and 1550 cm^−1^, associated with the presence of these functional groups. Variations in the pH of the solution revealed the ability of these groups to deprotonate or protonate, influencing the surface charge of CAC. At pH = 5.0, CAC acquires a predominantly negative charge, while the CIP and NOR molecules are predominantly cationic, favoring electrostatic interactions. Figure 7 presents a schematic of the potential adsorption mechanism of CIP and NOR on CAC.

In addition, the CAC surface contains aromatic functional groups that form double bonds between carbons (confirmed by FTIR), while CIP and NOR are organic compounds that have an aromatic structure. Therefore, CAC can exhibit π–π interactions with the CIP and NOR molecules, with CAC acting as the electron acceptor, while the aromatic ring of CIP and NOR is the donor [2]. The oxygenated groups of CAC can also form hydrogen bonds with the CIP and NOR molecules, owing to the highly electronegative elements present in the antibiotic molecules, or vice versa, possibly contributing to the adsorption mechanism [85,86]. 

The S_BET_ and well-developed pore structure of CAC (confirmed by the N_2_ adsorption/desorption isotherms and SEM analysis) provided abundant active sites for the adsorption of CIP and NOR. Furthermore, considering that the CIP and NOR molecules present a flat configuration and dimensions (length, width, and thickness) of approximately 13.5 Å × 3 Å × 7.4 Å in the case of CIP [87] and 13.28 Å × 2.56 Å × 7.54 Å for NOR [88], it is possible that they have easily accessed the pores of CAC, since the average pore diameter of CAC is 2.206 nm.

The kinetic, equilibrium, and thermodynamic analyses, together with the described potential adsorption mechanisms, indicate the predominance of physisorption and highlight the excellent performance of CAC in removing CIP and NOR from aqueous solutions. Such results not only broaden our understanding of the involved adsorption processes, but also reinforce the feasibility of using CAC as an effective adsorbent for removing CIP and NOR from aqueous systems.

### 2.9. Regeneration Study

**Figure 8:**
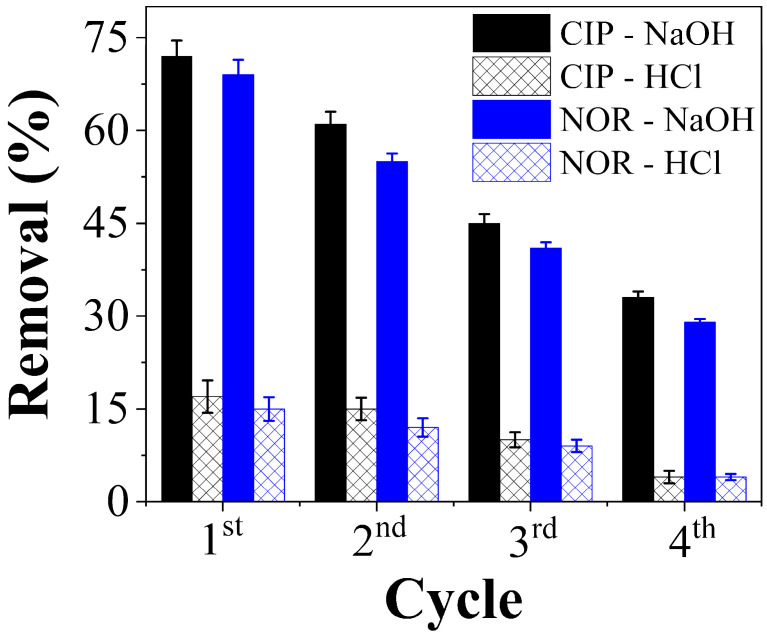
CAC regeneration tests using NaOH and HCl.

The results of the CAC regeneration tests using NaOH and HCl are shown in Figure 8. NaOH afforded the best results for both CIP and NOR removal after four adsorption/desorption cycles. The percentage of CIP removal with NaOH decreased from 72% in the first cycle to 33% at the end of the fourth cycle. In the case of NOR, the relevant value varied from 69% to 29%.

HCl afforded a relatively low removal efficiency, which was similar for both compounds and for each treatment cycle, rendering it unsuitable for the desorption of CAC. Traditionally, inorganic solvents such as NaOH and HCl have been successfully used to regenerate AC [89]. The choice of solvent depends on the nature of the initial interactions between the adsorbate and adsorbent, and the ways the regenerating solution affects the surface chemistry of the adsorbent and its possible interactions.

The results in Figure 8 show that NaOH was more effective in regenerating CAC, indicating that the existing electrostatic interactions play a dominant role in the adsorption of CIP and NOR. Changing the polarity of both the surface and the adsorbates was beneficial to the surface desorption process. This behavior is consistent with previously reported results indicating the superior desorption efficiency of NaOH in processes where physical mechanisms predominate, allowing for the chemical regeneration of the material and counteracting the present attractive forces [89,90].

Therefore, the reuse of CAC as an adsorbent for the removal of CIP and NOR from aqueous solutions is a viable and simple option. However, future studies are needed to evaluate its application on a large scale.

## 3. Materials and Methods

### 3.1. Preparation of AC

The cupuaçu peels were kindly donated by a fruit pulp company located in the Metropolitan Region of Belém, state of Pará. AC was obtained using the method reported by Chandrasekaran et al., with slight modifications [81]. The samples were washed with running water, sanitized with 10% sodium hypochlorite (NaClO), and dried at 80 °C in an oven with forced air circulation. They were then finely ground using a knife mill, washed with distilled water, and dried at 105 °C for 24 h. The biomass was activated with phosphoric acid (H_3_PO_4_, 88%) in a ratio of 1 g of biomass to 2.5 mL of acid solution at 60 °C for 6 h on a heating and stirring plate. After this stage, the acidified mixture was vacuum filtered, and the obtained samples were dried at 105 °C for 24 h. 

Carbonization was conducted in porcelain crucibles using approximately 5 g of sample at a time. The procedure was carried out in a muffle furnace under atmospheric air (QUIMIS Q-318M24, São Paulo, Brazil) at 600 °C for 2 h at a 17 °C/min heating rate and a heating gradient involving 1 h of heating at 300 °C, 1 h at 400 °C, and 1 h at 500 °C. The samples were repeatedly washed with 1% sodium bicarbonate (NaHCO_3_) to adjust the pH of distilled water, followed by drying at 80 °C for 24 h and storage in a hermetically sealed desiccator. The obtained AC was manually ground and sieved through a set of 10 to 12 mesh Tyler sieves, affording an average diameter of 1.40 mm. The samples were named granular activated carbon obtained from CAC and were stored in a hermetically sealed bag until further use in the adsorption experiments.

### 3.2. Characterization of AC

The CAC yield was calculated using Equation (8), where M*_initial_* is the initial mass of the biomass and $M_{final}$ is the obtained mass of carbon. The moisture and ash content were estimated using the gravimetric method in accordance with the ASTM D2867-23 and ASTM D2866-11 standards, respectively [22,91]. The yield, moisture, and ash tests were performed in triplicate, with the results presented as mean values ± standard deviations.

(8) $$R(\%)=\left(\frac{M_{initial}}{M_{final}}\right)\times 100$$

The pH of the surface of CAC was determined in accordance with ASTM 3838-05 [92]. The analysis of the surface functional groups was performed in triplicate using the neutralization and return volumetry technique, based on the Boehm method [93,94], which reveals the acidic and basic functional groups present on the surface of ACs. Standardized solutions of NaOH (0.1 N), Na_2_CO_3_ (0.1 N), and HCl (0.1 N) were used in all tests. Furthermore, the determination of the pH_PZC_ was based on the DRIFT method [18]. 

Suspensions containing 50 mg of CAC and 50 mL of a KCl solution (0.1 M) were prepared and their pH was adjusted between 2 and 12 using hydrochloric acid (HCl, 0.1 M) or sodium hydroxide (NaOH, 0.1 M). The suspensions were stirred on a shaker table rotating at 130 rpm at 28 °C for 24 h, after which, the pH of the filtered solutions was measured using a previously calibrated bench pH meter (PHS3BW model, Bel Engineering, Monza, Italy). Subsequently, the graph of the pH variation (∆*pH*) versus the initial pH of the samples was plotted to obtain the pH_PZC_ value.

The infrared spectrum of CAC was obtained using a FTIR (IRAffinity 1 model, Shimadzu, Tokyo, Japan) at a wavelength range between 400 and 4000 cm^−1^ and a resolution of 4 cm^−1^. The analysis was conducted using the KBr method and the results were measured in the transmittance mode. XRD was carried out on an X-Ray X’Pert-MPD diffractometer (PANalytical, Malvern, UK) using monochromatic copper radiation (Kα_1_ = 1.54 Å). The operating conditions were set at 2θ, 40 kV, and 40 mA.

The CAC nitrogen adsorption–desorption isotherms were obtained using a TRISTAR II 3020 surface area analyzer (Micromeritics, Norcross, GA, USA) to determine the S_BET_, volume, and pore size using the BET method. The samples underwent pre-treatment (degassing) at 300 °C for 3 h. The sorption/desorption isotherms were obtained under a nitrogen (N_2_) atmosphere at 77 K.

TG of CAC was carried out in a TGA-51H thermogravimetric analyzer (Shimadzu, Tokyo, Japan) using ~9 mg of the sample. During the analysis, the temperature varied from 30 °C (room temperature) to 1000 °C with a 10 °C/min heating rate, under a nitrogen atmosphere and with a flow rate of 50 mL/min. The morphology of CAC was investigated using SEM (model VEGA 3 TESCAN, Brno Czech Republic) with an electron beam current of 85–90 µA and an accelerating voltage of 10 kV. 

### 3.3. Preparation of Ciprofloxacin and Norfloxacin Solutions

High-purity CIP and NOR (≥98%, USP standard) from Sigma-Aldrich (Rio de Janeiro, Brazil) were used as adsorbates. The main physicochemical properties of these antibiotics are shown in Table S6. Two stock solutions of CIP and NOR (1000 mg/L) were prepared separately and were further diluted with distilled water to the concentrations required for our experiments. The CIP and NOR concentrations were determined using a UV–visible spectrophotometer (UV-1800, Shimadzu, Kyoto, Japan) at 270 and 273 nm, respectively. The maximum absorbance was experimentally verified via spectral scans in the range of 190 to 800 nm.

### 3.4. Batch Adsorption Tests

The adsorption tests were carried out in Erlenmeyer flasks (250 mL) with screw caps, using an orbital bath (Dubnoff-157, Solab, São Paulo, Brazil) with temperature control at a rotation of 130 rpm. Equations (9) and (10) were used to determine the adsorption capacity (*q*_*e*_) and percentage removal *R*(%) of each adsorbate, respectively.

(9) $$qe=\frac{V}{m}\left(C_{0}-C_{t}\right)$$

(10) $$Re(\%)=\frac{C_{0}-C_{t}}{C_{0}}\times 100$$

where *C*_0_ is the initial concentration (mg/L) of the solution, *C_t_* is the concentration at time *t* (mg/L), *m* is the mass of the adsorbent, and *V* represents the volume of the solution.

#### 3.4.1. Preliminary Tests 

To optimize the adsorption tests, preliminary tests were conducted to analyze the influence of pH and particle size of AC on the adsorption of CIP and NOR. To assess the influence of pH, solutions of HCl (0.1 M) and NaOH (0.1 M) were used to adjust the pH of the solution from 1.0 to 10.0, keeping the volume and concentration of the adsorbate fixed at 50 mL and 180 mg/L, respectively. Furthermore, the CAC particle size, adsorption time, CAC dosage, and temperature were kept stable at 1.4 mm, 240 min, 0.6 g/L, and 30 °C, respectively. The granulometry of CAC was evaluated using three different particle sizes, 1.80, 1.40, and 1.00 mm, based on previously reported studies [95] and ASTM D2862-16 [96]. The adsorption tests were conducted in triplicate, keeping the volume and concentration of the adsorbate (50 mL and 180 mg/L, respectively), adsorption time (240 min), solution pH (5.0), temperature (30 °C), and adsorbent dosage (0.6 g/L) stable.

#### 3.4.2. Experimental Design and Optimization

BBD was utilized to investigate the effects of the independent variables of the process (time, adsorbate concentration, and adsorbent dosage) on the adsorption capacity (*qe*) and removal percentage *R*(%). The actual and coded values of the input variables are presented in Table S7, and the standardized order design matrix is presented in Table S8, with the three-factor design requiring a combination of treatments. The BBD operating limits were chosen based on previously reported studies on the adsorption of other emerging contaminants on AC obtained from agro-industrial biomass [59,67]. 

A multiple linear regression (Equation (11)) was used to establish the relationship between the operational variables and the response variables, where $\hat{Y}$ corresponds to each of the response variables (dependent variables); *X*_1_, *X*_2_, and *X*_3_ are the coded levels of the operational variables (independent variables); *β*_0_ is a constant calculated from the intercept; and *β*_1_ to *β*_9_ are the linear, quadratic, and interaction regression coefficients between the terms [97].

(11) $$\hat{Y}=\beta_{0}+\beta_{1}X_{1}+\beta_{2}X_{2}+\beta_{3}X_{3}+\beta_{4}{X_{1}}^{2}+\beta_{5}{X_{2}}^{2}+\beta_{6}{X_{3}}^{2}+\beta_{7}X_{1}X_{2}+\beta_{8}X_{1}X_{3}+\beta_{9}X_{2}X_{3}$$

The verification of the fit of the proposed models was based on the R^2^ value resulting from the analysis of variance (ANOVA) and the analysis of the residuals (independence, normality, and homogeneity of the variance of the residuals). 

The desirability function has been used to simultaneously optimize multiple responses [68]. This technique comprises three basic steps: (1) definition of the interval between 0 (undesirable response) and 1 (desirable response) for η individual responses, (2) application of the conditions that correspond to various optimization results (the behavior of the function is directed when the individual response is maximized or minimized), and (3) construction of the global desirability function (D) (Equation (12)) by joining the individual desirabilities (*d_i_*) in such a way to afford the maximum global desirability (D) for m responses.

(12) $$D={\left(d_{i1}d_{i2}d_{i3}\ldots d_{im}\right)}^{1/m}$$

The objective was to maximize the adsorption capacities and removal percentages of the CIP-CAC and NOR-CAC systems. Statistical processing and analysis were carried out using Statistica^®^ 13.1 (DELL Inc., Santa Clara, CA, USA) with α = 0.05 as a level of significance.

### 3.5. Kinetic and Equilibrium Study

Adsorption kinetics and equilibrium studies were conducted in triplicate under the conditions indicated by the preliminary tests, determined by the desirability functions. The kinetic curves were obtained by measuring the concentration of CIP and NOR at predefined time intervals (2 to 300 min). Equilibrium curves were constructed by varying the initial concentrations of the adsorbates (2 to 330 mg/L) and measuring the adsorption capacity during 24 h of contact time at 28, 35, and 45 °C. Various mathematical models were fitted to the experimental kinetic and equilibrium data, as shown in Table S9.

To statistically evaluate the mathematical adjustments, the following statistics were used: coefficient of determination (*R*^2^), adjusted coefficient of determination ($R_{adjus}^{2}$), *RMSE*, and the corrected *AICc*, which are represented by Equations (13)–(16), respectively [98].

(13) $$R^{2}=\left[\frac{\sum_{i}^{n}{\left(q_{i,exp}-{\bar{q}}_{i,exp}\right)}^{2}-\sum_{i}^{n}{\left(q_{i,exp}-{\bar{q}}_{i,pred}\right)}^{2}}{\sum_{i}^{n}{\left(q_{i,exp}-{\bar{q}}_{i,exp}\right)}^{2}}\right]$$

(14) $$R_{adjus}^{2}=1-\left(1-R^{2}\right)-\frac{n-1}{p-1}$$

(15) $$RMSE=\sqrt{\sum_{i}^{n}\frac{{\left(q_{i,exp.}-q_{i,pred.}\right)}^{2}}{N}}$$

(16) $$AICc=nln\sum_{i}^{n}\left[\frac{{\left(q_{i,exp.}-q_{i,pred.}\right)}^{2}}{n}\right]+2\left(p+1\right)+\frac{2(p+1)(p+2)}{n-p-2}$$

where *n* is the number of experiments performed, *p* is the number of parameters in the fitted mathematical model, $q_{i,pred.}$ is the *q* value (adsorption capacity, in mg/g) predicted by the fitted mathematical model, ${\bar{q}}_{i,pred}$ is the average of these values, $q_{i,exp.}$ is the value observed experimentally, and ${\bar{q}}_{i,exp}$ is the average of the *q* values obtained in the experiments.

### 3.6. Adsorption Thermodynamics

The thermodynamic parameters were obtained at different temperatures (28, 35, and 45 °C). Gibbs free energy $\left(∆G^{0}\right)$ was calculated using Equation (17), while the enthalpy $\left(∆H^{0}\right)$ and entropy $\left({∆S}^{0}\right)$ were calculated using the Van’t Hoff equation (Equation (18)), which is obtained by combining the Gibbs free energy equation (Equation (17)) and the equation for the third principle of thermodynamics (Equation (19)) [99].

(17) $$∆G^{0}=-RTlnK_{e}^{0}$$

(18) $$LnK_{e}^{0}=-\frac{∆H^{0}}{R}\frac{1}{T}+\frac{{∆S}^{0}}{R}$$

(19) $$∆G^{0}=∆H^{0}-T{∆S}^{0}$$

where R is the universal gas constant (8314 J/mol K), T is the absolute temperature (K), and $K_{e}^{0}$ is the thermodynamic equilibrium constant (dimensionless). $∆H^{0}$ and ${∆S}^{0}$ are determined from the slope and intercept of the plot $LnK_{e}^{0}$ versus 1T, respectively. The units of $∆G^{0}$, $∆H^{0}$, and $∆S^{0}$ are J/mol, J/mol, and J/mol K, respectively [84]. The equilibrium constant $K_{e}^{0}$ was calculated as a function of the equilibrium constant *K*_*x*_, obtained directly from adjusting the isotherm, and the necessary considerations were made for its dimensioning, according to Wang et al. [79].

### 3.7. Regeneration Tests

The regeneration tests of the CAC saturated with CIP and NOR were conducted according to the methodology adapted from Wang et al. [89], using the NaOH (0.25 mol/L) and HCl (0.25 mol/L) desorption method. CIP and NOR were originally adsorbed onto CAC under the experimental conditions predicted by the Box–Behnken design and adsorption kinetics, aiming at fully occupying all the active sites on CAC before performing the regeneration tests. The CAC samples saturated with CIP and NOR were added separately to beakers containing 1000 mL of 0.25 mol/L NaOH and 1000 mL of 0.25 mol/L HCl for the desorption of CIP and NOR, followed by stirring at 125 rpm at room temperature. 

After desorption, CAC was separated from the NaOH and HCl solution, washed with distilled water, dried in an oven at 105 °C for 24 h, and submitted again to the adsorption cycle under the conditions predicted by the Box–Behnken design. The adsorption and desorption cycle were repeated four times to evaluate the CAC efficiency. In each cycle, the experiments were performed in triplicate, and the percentage removal results were expressed as means values ± standard deviations.

## 4. Conclusions

CAC proved to be effective in removing CIP and NOR from aqueous solutions, presenting favorable chemical and textural characteristics, such as a high quantity of acidic functional groups and a high specific surface area. The adsorption kinetics followed the PSO model and the good fit of the EMTR model indicated that external mass diffusion is the controlling step in the process. The Langmuir model fitted the experimental data best, indicating monolayer adsorption and favorable isotherms, with adsorption capacities of 6020 mg/g for CIP and 5701 mg/g for NOR. Thermodynamic analysis showed that increasing the temperature favors adsorption, suggesting endothermic processes and a predominance of physisorption. The adsorption mechanism mainly involved electrostatic forces between the CAC functional groups and the CIP and NOR molecules, as well as π–π interactions and hydrogen bonds. The regeneration tests indicated that NaOH is more effective than HCl, although the removal efficiency decreases after two cycles of use. Thus, the chemical and textural characteristics of CAC are favorable for the removal of the tested antibiotics, employing low-cost biomass and promoting the bioeconomy. Furthermore, the results of this study indicate the potential of incorporating CAC in water and wastewater treatment plants, as well as domestic filters, representing a significant advancement in the treatment of antibiotic-contaminated water. However, future studies are required to evaluate the application and optimization of the adsorbent on a large scale.

## Figures and Tables

**Table 1:** ANOVA results on the effects of the operational variables on each response.

Source	df	CIP-CAC						NOR-CAC					
		Removal			Adsorption Capacity			Removal			Adsorption Capacity		
		Coef	SS	*p*	Coef	SS	*p*	Coef	SS	*p*	Coef	SS	*p*
*X*_1_	1	6.07	294.52	0.0076 *	0.36	1.02	0.0026 *	6.54	342.30	0.0062 *	0.39	1.19	0.0039 *
*X*_2_	1	−9.66	745.75	0.0030 *	0.33	0.85	0.0031 *	−9.69	750.98	0.0028 *	0.29	0.68	0.0068 *
*X*_3_	1	5.23	218.82	0.0102 *	−0.28	0.64	0.0041 *	5.16	212.59	0.0099 *	−0.31	0.76	0.0060 *
$X_{1}^{2}$	1	−11.45	483.72	0.0046 *	−0.27	0.27	0.0097 *	−11.47	485.59	0.0044 *	−0.21	0.16	0.0272 *
$X_{2}^{2}$	1	−6.54	157.97	0.0140 *	−0.22	0.17	0.0149 *	−6.38	150.20	0.0140 *	−0.23	0.20	0.0223 *
$X_{3}^{2}$	1	−11.27	468.62	0.0048 *	−0.19	0.14	0.0184 *	−11.75	509.81	0.0042 *	−0.20	0.15	0.0291 *
*X*_1_ *X*_2_	1	−6.17	152.03	0.0145 *	0.17	0.11	0.0227 *	−5.58	124.66	0.0168 *	0.16	0.10	0.0424 *
*X*_1_ *X*_3_	1	2.39	22.75	0.0865	−0.16	0.10	0.0248 *	2.08	17.31	0.1049	−0.19	0.14	0.0306 *
*X*_2_ *X*_3_	1	−2.25	20.25	0.0957	−0.04	0.01	0.2395	−1.79	12.82	0.1345	−0.10	0.04	0.0948
Lack of fit	3		35.26	0.1653		0.03	0.2029		37.98			0.01	
Pure error	2		4.51			0.01			4.29			0.01	
Total	14		2468.55			3.27			2510.39			3.38	
$\beta_{0}$		95.29		<0.0001 *	5.81		<0.0001 *	92.99		<0.0001 *	5.54		<0.0001 *
R^2^		0.9839			0.9885			0.9832			0.9956		

df: degrees of freedom; Coef.: regression coefficient; SS: sum of squares; $\beta_{0}$: intercept; *p*: probability of significance; * statistically significant values (*p* ≤ 0.05).

**Table 2:** Conditions predicted by the desirability function and experimentally determined results.

Optimized Adsorption Conditions	CIP-CAC	NOR-CAC
Time (min.)	266.40	273.60
Initial adsorbate concentration (mg/L)	192	186
Adsorbent dosage (g/L)	0.57	0.55
Global desirability	0.993	0.989
Predicted values		
Removal (%)	93.44	90.36
*qe* (mg/g)	6.00	5.80
Experimental values *		
Removal (%)	92.89 ± 2.56	89.21 ± 2.35
*qe* (mg/g)	5.90 ± 0.16	5.71 ± 0.19

* performed in triplicate; values expressed as mean ± standard deviation.

**Table 3:** Adjustment of the PPO, PSO, and Elovich models to the experimental data of the CIP and NOR adsorption on CAC.

Models	Parameters	CIP-CAC	NOR-CAC
Experimental *	$q_{e}$ (mg/g)	5.901 ± 0.275	5.682 ± 0.201
PPO	$q_{e}$ (mg/g)	5.511	5.287
	$k_{1}$ (min^−1^)	0.045	0.054
	*R* ^2^	0.977	0.969
	*R* ^2^_ajus_	0.976	0.968
	*RMSE*	0.291	0.316
	*AIC_C_*	−80.22	−75.43
PSO	$q_{e}$ (mg/g)	6.172	5.862
	$k_{2}$ (min^−1^)	0.009	0.012
	*R* ^2^	0.998	0.996
	*R* ^2^ *_ajus_*	0.998	0.996
	*RMSE*	0.089	0.113
	*AIC_C_*	−90.47	−86.69
Elovich	α (mg/g min)	0.798	1.056
	β (mg/g)	0.841	0.936
	*R* ^2^	0.979	0.975
	*R* ^2^ *_ajus_*	0.978	0.973
	*RMSE*	0.277	0.286
	*AIC_C_*	−50.89	−43.21

* performed in triplicate; values expressed as mean ± standard deviation. *RMSE*: root mean square error; *AICc*: Akaike information criterion; *R*^2^: coefficient of determination.

**Table 4:** Fitting of the Langmuir, Freundlich, and Sips models to the adsorption equilibrium data.

Models	Parameters	CIP-CAC			NOR-CAC		
		28 °C	35 °C	45 °C	28 °C	35 °C	45 °C
Experimental *	$q_{m}$ (mg/g)	6.020 ± 0.192	6.050 ± 0.199	6.130 ± 0.202	5.701 ± 0.181	5.790 ± 0.190	5.850 ± 0.195
Langmuir	$q_{m}$ (mg/g)	6.153	6.169	6.189	5.984	5.999	6.016
	$K_{L}$ (L/mg)	0.073	0.080	0.090	0.046	0.050	0.054
	*R* ^2^	0.966	0.965	0.967	0.967	0.964	0.962
	*R* ^2^ *_ajus_*	0.962	0.961	0.963	0.963	0.963	0.958
	*RMSE*	0.313	0.310	0.297	0.318	0.328	0.334
	*AICc*	−80.22	−79.92	−80.89	−81.45	−80.96	−80.03
Freundlich	$K_{F}$ [(mg/g)(L/mg)^1/n^_F_]	1.732	1.834	1.950	1.333	1.421	1.500
	*n*	4.438	4.619	4.820	3.812	3.949	4.073
	*R* ^2^	0.947	0.944	0.945	0.962	0.960	0.959
	*R* ^2^ *_ajus_*	0.942	0.934	0.939	0.958	0.956	0.956
	*RMSE*	0.386	0.393	0.384	0.340	0.342	0.343
	*AICc*	−71.57	−65.87	−66.99	−80.86	−79.44	−79.32
Sips	$q_{m}$ (mg/g)	7.155	7.096	7.081	7.123	7.223	7.241
	$K_{s}$ [(L/mg)^1/*ns*^]	0.149	0.160	0.177	0.105	0.116	0.125
	*n_s_*	1.585	1.585	1.585	1.548	1.591	1.608
	*R* ^2^	0.988	0.989	0.992	0.994	0.993	0.993
	*R* ^2^ *_ajus_*	0.985	0.986	0.991	0.992	0.991	0.992
	*RMSE*	0.193	0.185	0.146	0.147	0.156	0.148
	*AICc*	−85.44	−87.33	−91.39	−90.63	−89.92	−91.24

* performed in triplicate; values expressed as mean ± standard deviation.

**Table 5:** Estimated thermodynamic parameters for the adsorption of CIP and NOR on CAC.

	CIP–CAC			NOR–CAC		
	28 °C	35 °C	45 °C	28 °C	35 °C	45 °C
*K_L_* (L/mg)	0.073	0.080	0.090	0.046	0.050	0.054
$K_{e}^{0}$ (adim.)	24,187.82	26,507.20	29,820.6	10,089.18	10,966.50	11,843.82
ΔG° (kJ/mol)	−25.27	−26.09	−27.25	−14.88	−15.64	−16.56
ΔH° (kJ/mol)	9.78			7.37		
ΔS° (J/mol K)	116.41			101.18		
*R* ^2^	0.999			0.988		
*R* ^2^ *_ajus_*	0.998			0.977		

## Data Availability

The data presented in this study are available upon request from the corresponding authors.

## Supplementary Materials

The following supporting information can be downloaded at: https://www.mdpi.com/article/10.3390/molecules29245853/s1, Table S1. Characterization of activated carbons prepared from different precursors and activating agents; Table S2. BBD experiment matrix; Table S3. Parameters assumed in the global desirability function; Table S4. Separation factor (RL) calculated on the CIP and NOR isotherms in CAC; Table S5 Maximum adsorption capacity of CIP and NOR on CAC compared to other adsorbents; Table S6. The main physicochemical properties of Ciprofloxacin (CIP) and Norfloxacin (NOR); Table S7. Operational variables and levels of Box–Behnken Design; Table S8. Standardized order design matrix; Table S9. Kinetic and isothermal models; Figure S1. Fitting of the Langmuir model to the experimental adsorption equilibrium data for CIP (a) and NOR (b) on CAC. These references [2,7,18,28,35,43,66,85,87,89,100,101,102,103,104,105,106,107,108,109,110,111,112,113,114,115,116,117] are cited in Supplementary Materials.
