# Supplementary material for: Removal of Ciprofloxacin and Norfloxacin from Aqueous Solution with Activated Carbon from Cupuaçu (Theobroma grandiflorum) Bark
Source: Molecules. 2024 Dec 11;29(24):5853. doi: 10.3390/molecules29245853 (PMC11676745; doi:10.3390/molecules29245853)
Supplement: Supplementary file 1 [file molecules-29-05853-s001.zip › molecules-3298921-supplementary.pdf]

Removal of Ciprofloxacin and Norfloxacin from Aqueous Solution with Activated Carbon from Cupuaçu (*Theobroma grandiflorum*) Bark

Rafael Alves do Nascimento [1,2,*], Nilson dos Reis de Oliveira Novaes [2], Demetrius Pereira Morilla [3], Patricia Teresa Souza da Luz [4], Cristiane Maria Leal Costa [5,6] and Lênio José Guerreiro de Faria [1,5,6]

[1] Postgraduate Program in Amazonian Natural Resources Engineering, Federal University of Pará, Rua Augusto Correa, 01, Belém 66075-110, Brazil; lenio@ufpa.br
[2] Federal Institute of Education Science and Technology of Rondônia, Porto Velho Calama Campus, Av. Calama, 4985, Porto Velho 76820-441, Brazil; nilson.novaes@ifro.edu.br
[3] Federal Institute of Education Science and Technology of Alagoas, Maceió Campus, Rua Mizael Domingues, 530, Maceió 57020-600, Brazil; demetrius.morilla@ifal.edu.br
[4] Federal Institute of Education Science and Technology of Pará, Belém Campus, Av. Alm. Barroso, 1155, Belém 66093-020, Brazil; patricia.luz@ifpa.edu.br
[5] Postgraduate Program in Chemical Engineering, Federal University of Pará, Rua Augusto Correa, 01, Belém 66075-110, Brazil; cristianemlcosta@gmail.com
[6] Chemical Engineering School, Federal University of Pará, Rua Augusto Correa, 01, Belém 66075-110, Brazil
* Correspondence: rafaelnascimentoa@gmail.com; Tel.: +55-91984927951

Supporting Information for the manuscript:

Tables

Table S1. Characterization of activated carbons prepared from different precursors and activating agents

Activated carbon	Activating agente	S_{BET} (m^2/g)	V_T (cm^3/g)	D_p (nm)	References
Cupuaçu bark	H_3PO_4	1335.66	0.753	2.206	This study
Brazil nut shell	H_3PO_4	1651.31	1.194	-	[1]
Açaí stone	H_3PO_4	990.81	0.722	-	[1]
Green coconut shell	KOH	1005.77	0.653	-	[2]
Corn stigmata fibers	KOH	11	0.010	563.2	[3]
Corn stigmata fibers	H_3PO_4	589	0.280	10.2	[3]
Corn stigmata fibers	$ZnCl_2$	389	0.180	15.4	[3]
Waste murumuru	$ZnCl_2$	271.18	0.108	0.436	[4]
Cistus shells	H_3PO_4	1210	0.750	2.730	[5]
Macadamia nut shells	H_3PO_4	1190	1.220	-	[6]

Table S2. BBD experiment matrix

Tests	Factors			CIP-CAC		NOR-CAC	
	Time (X_1)	Concentration (X_2)	Dosage (X_3)	Re (%)	qe (mg/g)	Re (%)	qe (mg/g)
1	-1	-1	0	72.22	4.85	70.12	4.61
2	1	-1	0	98.5	5.23	96.22	5.01
3	-1	1	0	68.43	5.09	65.24	4.87
4	1	1	0	70.05	6.14	69.01	5.91
5	-1	0	-1	65.59	5.17	62.14	4.83
6	1	0	-1	71.14	6.2	69.21	6.03
7	-1	0	1	69.24	4.82	66.18	4.61
8	1	0	1	84.33	5.21	81.57	5.05
9	0	-1	-1	80.23	5.23	78.22	5.03
10	0	1	-1	62.23	6.04	59.09	5.82
11	0	-1	1	97.23	4.85	94.22	4.6
12	0	1	1	70.23	5.49	67.93	4.98
13	0	0	0	95.55	5.77	93.03	5.52
14	0	0	0	93.67	5.87	91.51	5.62
15	0	0	0	96.64	5.8	94.44	5.49

Table S3. Parameters assumed in the global desirability function

Adsorbent-Adsorbate	Response variables	Parameters assumed in optimization				
		Low	Medium	Higth	s	t
CAC-CIP	Removal (% Re)	62.23 (0)	80.36 (0)	98.5 (1)	4	4
	Adsorption capacity (qe)	4.82 (0)	5.51 (0)	6.20 (1)	4	4
CAC-NOR	Removal (% Re)	59.09 (0)	77.65 (0)	96.22 (1)	4	4
	Adsorption capacity (qe)	4.60 (0)	5.31(0)	6.03 (1)	4	4

(0) Unacceptable values. (1) Acceptable values for the desirability function. s and t: Exponents of the desirability function.

Table S4. Separation factor (R_L) calculated on the CIP and NOR isotherms in CAC

C_0 (mg/L)	CIP-CAC			NOR-CAC		
	28 ºC	35 ºC	45º C	28 ºC	35 ºC	45 ºC
2	0.873	0.862	0.847	0.916	0.909	0.903
8	0.631	0.610	0.581	0.731	0.714	0.698
15	0.477	0.455	0.426	0.592	0.571	0.552
30	0.313	0.294	0.270	0.420	0.400	0.382
50	0.215	0.200	0.182	0.303	0.286	0.270
100	0.120	0.111	0.100	0.179	0.167	0.156
150	0.084	0.077	0.069	0.127	0.118	0.110
200	0.064	0.059	0.053	0.098	0.091	0.085
250	0.052	0.048	0.043	0.080	0.074	0.069
300	0.044	0.040	0.036	0.068	0.063	0.058
330	0.040	0.036	0.033	0.062	0.057	0.053

Table S5. Maximum adsorption capacity of CIP and NOR on CAC compared to other adsorbents.

Adsorbent	qm (mg/g)	pH of the solution	Temperature (º C)	Adsorbate	Reference
CAC	6.02	5.0	28	CIP	This study
CAC	5.70	5.0	28	NOR	This study
Oryza sativa husk ash	7.47	8.0	25	CIP	[7]
Carbon Nanotubes	1.74	-	-	CIP	[8]
Commercial AC	1.86	-	25	CIP	[9]
Montmorillonite	0.60	-	25	CIP	[9]
Granular AC from date seed	2.09	9.0	30	CIP	[10]
Carbon nanofibers	10.36	5.0	25	CIP	[11]
Granular AC from activated sludge	13.78	7.0	28	CIP	[12]
Magnetized AC from willow	6.26	-	25	NOR	[13]
Granular AC from date seed	1.99	5.0	30	NOR	[10]
Nanocomposite of CA/$Fe_2 0_3$/β cyclodextrin and sodium alginate	2.55	5.0	35	NOR	[14]
Biochar loaded with manganese oxide	6.25	7.0	25	NOR	[15]
Iron ore waste	6.48	4.0	25	NOR	[16]
Biochar from grapefruit peels	3.75	3.0	45	NOR	[17]

Table S6. The main physicochemical properties of Ciprofloxacin (CIP) and Norfloxacin (NOR)

Properties	Ciprofloxacin	Norfloxacin	References
Molecular fórmula	$C_{17}H_{18}FN_3O_3$	$C_{16}H_{18}FN_3O_3$	[18]
Wave-length (λ, nm)	270	273	[18]
Molecular weight (g/mol)	331.34	319.33	[18]
Water solubility (mg/mL)	3.46	161	[19]
pKa_1 e pKa_2	6.1 e 8.7	6.1 e 8.3	[18]
CAS number	85721-33-1	70458-96-7	[18]

Table S7. Operational variables and levels of Box-Behnken Desing.

Original Variables	Coded variables	Units	Levels		
			-1	0	+1
Contact time (t)	X_1	(min.)	180	240	300
Adsorbate concentration (Cant)	X_2	(mg/L)	30	180	330
Adsorbent dosage (Ccac)	X_3	(g/L)	0.4	0.6	0.8

Table S8. Standardized order desing matrix

Tests	Factors		
	X_1	X_2	X_3
1	-1	-1	0
2	1	-1	0
3	-1	1	0
4	1	1	0
5	-1	0	-1
6	1	0	-1
7	-1	0	1
8	1	0	1
9	0	-1	-1
10	0	1	-1
11	0	-1	1
12	0	1	1
13	0	0	0
14	0	0	0
15	0	0	0

Table S9. Kinetic and isothermal models.

Kinetic models	Nonlinear equation	Reference
Pseudo-first order	$q_t = q_e[1 - \exp(-k_1 t)]$	[20]
Pseudo-second order	$q_t = \frac{q_e^2 k_2 t}{1 + q_e k_2 t}$	[21]
Elovich	$q_t = \frac{1}{\beta} ln(1 + \alpha\beta t)$	[22]
Intraparticle diffusion	$q_t = k_{dif} t^{0,5} + C_d$	[23]
Boyd	$F = \frac{q_t}{q_e}$<br>$Bt = -ln\frac{\pi^2}{6} - \ln(1 - F(t))$ for F(t)>0,85<br>$Bt = \left(\sqrt{\pi} - \sqrt{\pi - \frac{\pi^2 F(t)}{3}}\right)^2$ for F(t)≤0,85	[24]
EMTR	$\frac{dC_t}{dt} = -K_{TM}\,(C_{(t)} - C_s)$<br>$\frac{dC_s}{dt} = \frac{VK_{TM}}{mq_m K_L}[1 + K_L C_s]^2[C_{(t)} - C_s]$	[25]

Isotherms models	Nonlinear equation	Reference
Langmuir	$q_e = \dfrac{q_m K_L C_e}{1 + K_L C_e}$	[26]
Freundlich	$q_e = K_F C_e^{(1/n_F)}$	[27]
Sips	$q_e = \dfrac{q_m K_S C_e^{(1/n_S)}}{1 + K_S C_e^{(1/n_S)}}$	[28]

where q_e and q_t amount of adsorbate adsorbed at equilibrium and at any time (mg/g); k_1 and k_2: pseudo-first order (1/min) and pseudo-second-order (g/mg min) constants; α and β: respectively the initial adsorption rate (mg/g min) and the desorption constant (mg/g) of the Elovich model; k_{dif}: intraparticle diffusion rate constant (mg/g min$^{0.5}$); C: parameter related to the boundary layer thickness; F: fraction of solute adsorbed at time t; Bt: mathematical function of F; K_{TM}: is the mass transfer coefficient in external film (m/s); V is the volume of adsorbate solution (L); m is the mass of the adsorbent (g); $C(t)$ is the concentration of adsorbate within the solution at time t (mg/g); Cs is the adsorbate concentration at the interface liquid-solid (mg/L); K_L: affinity constant between adsorbent and adsorbate (L/mg) q_m: maximum adsorption capacity (mg/g); Ce: equilibrium concentration of adsorbate in solution (mg/L); K_F: Freundich's constant [(mg/g) (L/mg)$^{1/n_F}$]; n_F: Freundlich heterogeneity factor; K_S: Sips constant (L/mg)$^{1/ns}$; n_S: Sips factor.

FIGURES

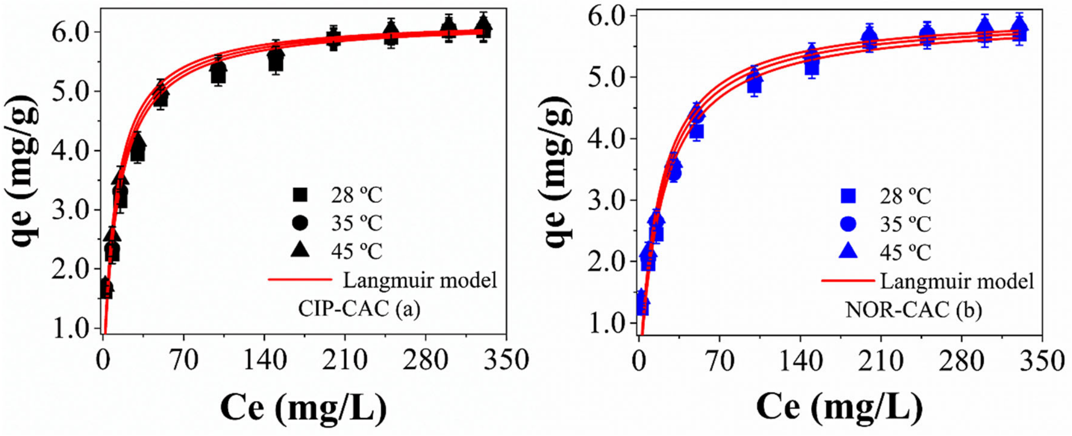

Figure S1. Fitting of the Langmuir model to the experimental adsorption equilibrium data for CIP (a) and NOR (b) on CAC.

References

1. De Souza, T.N.V.; Vieira, M.G.A.; Da Silva, M.G.C.; Brasil, D.D.S.B.; De Carvalho, S.M.L. H3PO4-Activated Carbons Produced from Açai Stones and Brazil Nut Shells: Removal of Basic Blue 26 Dye from Aqueous Solutions by Adsorption. *Environ Sci Pollut Res* **2019**, *26*, 28533–28547, doi:10.1007/s11356-019-04215-0.
2. DasSharma, D.; Samanta, S.; S, D.N.K.; Halder, G. A Mechanistic Insight into Enrofloxacin Sorptive Affinity of Chemically Activated Carbon Engineered from Green Coconut Shell. *Journal of Environmental Chemical Engineering* **2020**, *8*, 104140, doi:10.1016/j.jece.2020.104140.
3. Mbarki, F.; Selmi, T.; Kesraoui, A.; Seffen, M. Low-Cost Activated Carbon Preparation from Corn Stigmata Fibers Chemically Activated Using H3PO4, ZnCl2 and KOH: Study

of Methylene Blue Adsorption, Stochastic Isotherm and Fractal Kinetic. *Industrial Crops and Products* **2022**, *178*, 114546, doi:10.1016/j.indcrop.2022.114546.

4. Costa, R.L.T.; Do Nascimento, R.A.; De Araújo, R.C.S.; Vieira, M.G.A.; Da Silva, M.G.C.; De Carvalho, S.M.L.; De Faria, L.J.G. Removal of Non-Steroidal Anti-Inflammatory Drugs (NSAIDs) from Water with Activated Carbons Synthetized from Waste Murumuru (Astrocaryum Murumuru Mart.): Characterization and Adsorption Studies. *Journal of Molecular Liquids* **2021**, *343*, 116980, doi:10.1016/j.molliq.2021.116980.
5. El Farissi, H.; Beraich, A.; Lamsayah, M.; Talhaoui, A.; El Bachiri, A. The Efficiency of Carbon Modified by Phosphoric Acid (H3PO4) Used in the Removal of Two Antibiotics Amoxicillin and Metronidazole from Polluted Water: Experimental and Theoretical Investigation. *Journal of Molecular Liquids* **2023**, *391*, 123237, doi:10.1016/j.molliq.2023.123237.
6. Harabi, S.; Guiza, S.; Álvarez-Montero, A.; Gómez-Avilés, A.; Belver, C.; Rodríguez, J.J.; Bedia, J. Adsorption of 2,4-Dichlorophenoxyacetic Acid on Activated Carbons from Macadamia Nut Shells. *Environmental Research* **2024**, *247*, 118281, doi:10.1016/j.envres.2024.118281.
7. Dhiman, N. Analysis of Non Competitive and Competitive Adsorption Behaviour of Ciprofloxacin Hydrochloride and Ofloxacin Hydrochloride from Aqueous Solution Using Oryza Sativa Husk Ash (Single and Binary Adsorption of Antibiotics). *Cleaner Materials* **2022**, *5*, 100108, doi:10.1016/j.clema.2022.100108.
8. Avcı, A.; İnci, İ.; Baylan, N. Adsorption of Ciprofloxacin Hydrochloride on Multiwall Carbon Nanotube. *Journal of Molecular Structure* **2020**, *1206*, 127711, doi:10.1016/j.molstruc.2020.127711.
9. Avcı, A.; İnci, İ.; Baylan, N. A Comparative Adsorption Study with Various Adsorbents for the Removal of Ciprofloxacin Hydrochloride from Water. *Water Air Soil Pollut* **2019**, *230*, 250, doi:10.1007/s11270-019-4315-6.
10. Darweesh, T.M.; Ahmed, M.J. Adsorption of Ciprofloxacin and Norfloxacin from Aqueous Solution onto Granular Activated Carbon in Fixed Bed Column. *Ecotoxicology and Environmental Safety* **2017**, *138*, 139–145, doi:10.1016/j.ecoenv.2016.12.032.
11. Li, X.; Wang, W.; Dou, J.; Gao, J.; Chen, S.; Quan, X.; Zhao, H. Dynamic Adsorption of Ciprofloxacin on Carbon Nanofibers: Quantitative Measurement by in Situ Fluorescence. *Journal of Water Process Engineering* **2016**, *9*, e14–e20, doi:10.1016/j.jwpe.2014.12.006.
12. Gupta, A.; Garg, A. Adsorption and Oxidation of Ciprofloxacin in a Fixed Bed Column Using Activated Sludge Derived Activated Carbon. *Journal of Environmental Management* **2019**, *250*, 109474, doi:10.1016/j.jenvman.2019.109474.
13. Wang, M.; Li, G.; Huang, L.; Xue, J.; Liu, Q.; Bao, N.; Huang, J. Study of Ciprofloxacin Adsorption and Regeneration of Activated Carbon Prepared from Enteromorpha Prolifera Impregnated with H 3 PO 4 and Sodium Benzenesulfonate. *Ecotoxicology and Environmental Safety* **2017**, *139*, 36–42, doi:10.1016/j.ecoenv.2017.01.006.
14. Yadav, S.; Asthana, A.; Singh, A.K.; Chakraborty, R.; Vidya, S.S.; Susan, Md.A.B.H.; Carabineiro, S.A.C. Adsorption of Cationic Dyes, Drugs and Metal from Aqueous Solutions Using a Polymer Composite of Magnetic/β-Cyclodextrin/Activated Charcoal/Na Alginate: Isotherm, Kinetics and Regeneration Studies. *Journal of Hazardous Materials* **2021**, *409*, 124840, doi:10.1016/j.jhazmat.2020.124840.
15. Liao, X.; Chen, C.; Liang, Z.; Zhao, Z.; Cui, F. Selective Adsorption of Antibiotics on Manganese Oxide-Loaded Biochar and Mechanism Based on Quantitative Structure–Property Relationship Model. *Bioresource Technology* **2023**, *367*, 128262, doi:10.1016/j.biortech.2022.128262.

16. Fang, N.; He, Q.; Sheng, L.; Xi, Y.; Zhang, L.; Liu, H.; Cheng, H. Toward Broader Applications of Iron Ore Waste in Pollution Control: Adsorption of Norfloxacin. *Journal of Hazardous Materials* **2021**, *418*, 126273, doi:10.1016/j.jhazmat.2021.126273.
17. Zhang, L.-L.; Chen, Y.; Li, Z.-J.; Li, X.; Fan, G. Bioactive Properties of the Aromatic Molecules of Spearmint (Mentha Spicata L.) Essential Oil: A Review. *Food Funct.* **2022**, *13*, 3110–3132, doi:10.1039/D1FO04080D.
18. PubChem National Library of Medicine: National Center for Biotechnology Information (NCBI). DataBase Pubchem DataBase. Ciprofloxacin and Norfloxacin.
19. Ashiq, A.; Vithanage, M.; Sarkar, B.; Kumar, M.; Bhatnagar, A.; Khan, E.; Xi, Y.; Ok, Y.S. Carbon-Based Adsorbents for Fluoroquinolone Removal from Water and Wastewater: A Critical Review. *Environmental Research* **2021**, *197*, 111091, doi:10.1016/j.envres.2021.111091.
20. Lagergren, S. Zur Theorie Der Sogenannten Adsorption Gelöster Stoffe Kungliga Svenska Vetenskapsakademien. *Handlingar* **1898**, *24*, 1–39.
21. Ho, Y.S.; McKay, G. Sorption of Dye from Aqueous Solution by Peat. *Chemical Engineering Journal* **1998**, *70*, 115–124, doi:10.1016/S0923-0467(98)00076-1.
22. Aharoni, C.; Tompkins, F.C. Kinetics of Adsorption and Desorption and the Elovich Equation. In *Advances in Catalysis*; Eley, D.D., Pines, H., Weisz, P.B., Eds.; Academic Press, 1970; Vol. 21, pp. 1–49.
23. Weber, W.J.; Morris, J.C. Kinetics of Adsorption on Carbon from Solution. *Journal of the Sanitary Engineering Division* **1963**, *89*, 31–59, doi:10.1061/JSEDAI.0000430.
24. Boyd, G.E.; Adamson, A.W.; Myers, L.S.Jr. The Exchange Adsorption of Ions from Aqueous Solutions by Organic Zeolites. II. Kinetics1. *J. Am. Chem. Soc.* **1947**, *69*, 2836–2848, doi:10.1021/ja01203a066.
25. Puranik, P.R.; Modak, J.M.; Paknikar, K.M. A Comparative Study of the Mass Transfer Kinetics of Metal Biosorption by Microbial Biomass. *Hydrometallurgy* **1999**, *52*, 189–197, doi:10.1016/S0304-386X(99)00017-1.
26. Langmuir, I. The Adsorption of Gases on Plane Surfaces of Glass, Mica and Platinum. *Journal of the American Chemical Society* **1918**, *40*, 1361–1403, doi:https://doi.org/10.1021/ja02242a004.
27. Freundlich, H.M.F. Over the Adsorption in Solution. *Journal of Physical Chemistry* **1906**, 1100–1107.
28. Sips, R. On the Structure of a Catalyst Surface. *The Journal of Chemical Physics* **1948**, *16*, 490–495, doi:10.1063/1.1746922.
